# Supplementary material for: A hexa-species transcriptome atlas of mammalian embryogenesis delineates metabolic regulation across three different implantation modes
Source: Nat Commun. 2022 Jun 16;13:3407. doi: 10.1038/s41467-022-30194-x (PMC9203550; doi:10.1038/s41467-022-30194-x)
Supplement: Supplementary file 1 — Supplementary Information [file 41467_2022_30194_MOESM1_ESM.pdf]

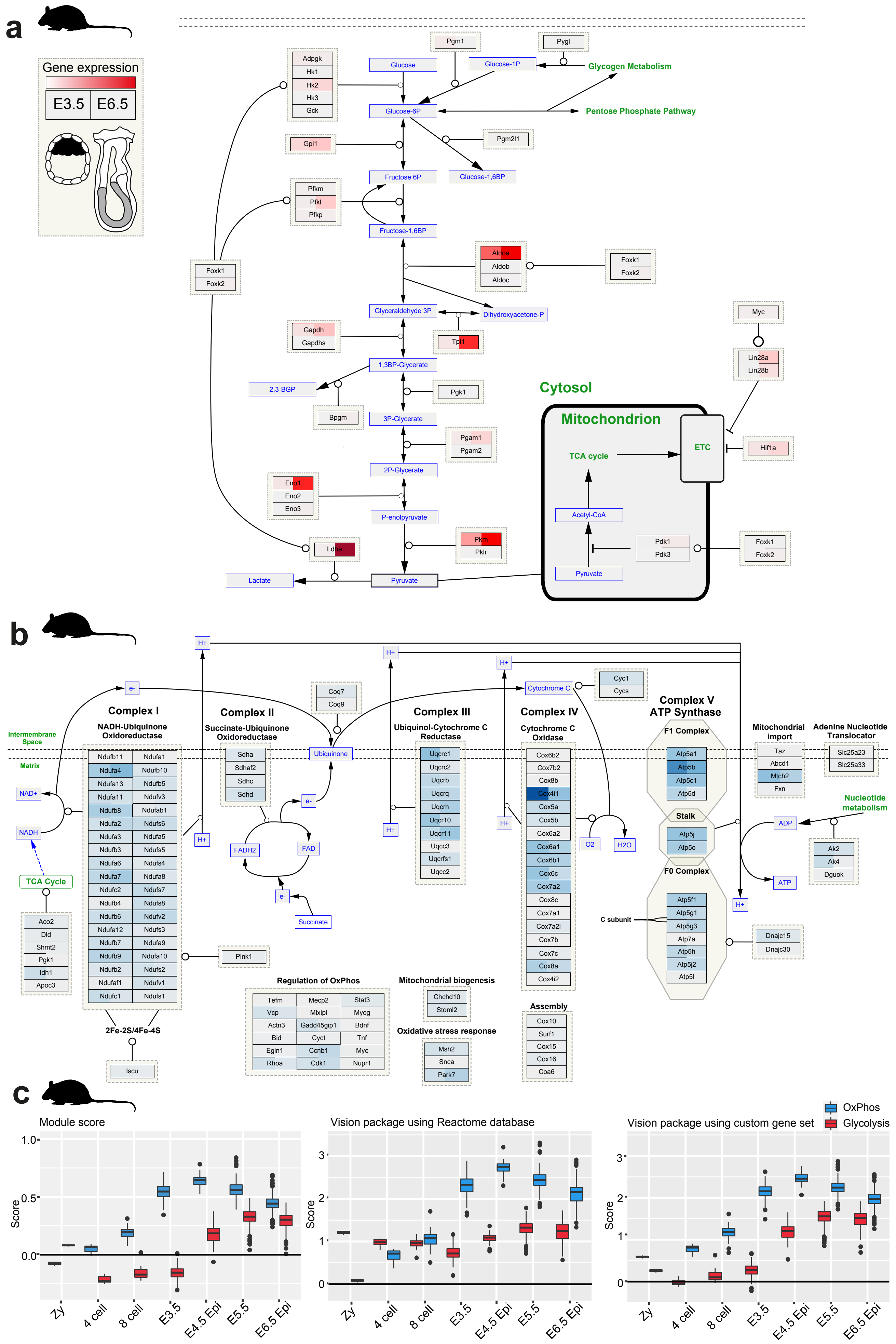
a
Gene expression
E3.5
E6.5
Glycogen Metabolism
Pentose Phosphate Pathway
Pgm1
Pygl
Adpgk
Hk1
Hk2
Hk3
Gck
Glucose
Glucose-1P
Glucose-6P
Gpi1
Pgm2l1
Fructose 6P
Glucose-1,6BP
Pfkm
Pfkl
Pfkp
Fructose-1,6BP
Foxk1
Foxk2
Aldoa
Aldob
Aldoc
Glyceraldehyde 3P
Dihydroxyacetone-P
Gapdh
Gapdhs
Tpi1
1,3BP-Glycerate
2,3-BGP
Bpgm
Pgk1
3P-Glycerate
Pgam1
Pgam2
2P-Glycerate
Eno1
Eno2
Eno3
P-enolpyruvate
Pkm
Pklr
Ldha
Lactate
Pyruvate
Myc
Lin28a
Lin28b
Cytosol
Mitochondrion
TCA cycle
ETC
Hif1a
Acetyl-CoA
Pdk1
Pdk3
b
Intermembrane Space
Matrix
H+
e-
Complex I
NADH-Ubiquinone Oxidoreductase
Ndufb11
Ndufa1
Ndufa4
Ndufb10
Ndufa13
Ndufb5
Ndufa11
Ndufv3
Ndufb8
Ndufab1
Ndufa2
Ndufs6
Ndufa3
Ndufa5
Ndufb3
Ndufs5
Ndufa6
Ndufs4
Ndufa7
Ndufa8
Ndufc2
Ndufs7
Ndufb4
Ndufs8
Ndufb6
Ndufv2
Ndufa12
Ndufs3
Ndufb7
Ndufa9
Ndufb9
Ndufa10
Ndufb2
Ndufs2
Ndufaf1
Ndufv1
Ndufc1
Ndufs1
NAD+
NADH
TCA Cycle
Aco2
Dld
Shmt2
Pgk1
Idh1
Apoc3
Pink1
2Fe-2S/4Fe-4S
Iscu
Complex II
Succinate-Ubiquinone Oxidoreductase
Sdha
Sdhaf2
Sdhc
Sdhd
Coq7
Coq9
Ubiquinone
FADH2
FAD
Succinate
Complex III
Ubiquinol-Cytochrome C Reductase
Uqcrc1
Uqcrc2
Uqcrb
Uqcrq
Uqcrh
Uqcr10
Uqcr11
Uqcc3
Uqcrfs1
Uqcc2
Cytochrome C
Cyc1
Cycs
Complex IV
Cytochrome C Oxidase
Cox6b2
Cox7b2
Cox8b
Cox4i1
Cox5a
Cox5b
Cox6a2
Cox6a1
Cox6b1
Cox6c
Cox7a2
Cox8c
Cox7a1
Cox7a2l
Cox7b
Cox7c
Cox8a
Cox4i2
O2
H2O
Assembly
Cox10
Surf1
Cox15
Cox16
Coa6
Complex V
ATP Synthase
F1 Complex
Atp5a1
Atp5b
Atp5c1
Atp5d
Stalk
Atp5j
Atp5o
F0 Complex
Atp5f1
Atp5g1
Atp5g3
Atp7a
Atp5h
Atp5j2
Atp5l
C subunit
Dnajc15
Dnajc30
Mitochondrial import
Taz
Abcd1
Mtch2
Fxn
Adenine Nucleotide Translocator
Slc25a23
Slc25a33
Nucleotide metabolism
ADP
ATP
Ak2
Ak4
Dguok
Regulation of OxPhos
Tefm
Mecp2
Stat3
Vcp
Mlxipl
Myog
Actn3
Gadd45gip1
Bdnf
Bid
Cyct
Tnf
Egln1
Ccnb1
Myc
Rhoa
Cdk1
Nupr1
Mitochondrial biogenesis
Chchd10
Stoml2
Oxidative stress response
Msh2
Snca
Park7
c
Module score
Vision package using Reactome database
Vision package using custom gene set
OxPhos
Glycolysis
Score
1.0
0.5
0.0
3
2
1
0
Zy
4 cell
8 cell
E3.5
E4.5 Epi
E5.5
E6.5 Epi

Supplementary Figure 1

(a) Network of glycolysis-related genes used to calculate the module score for the mouse dataset. Relative gene expression in the epiblast at E3.5 and E6.5 is visualised by the colour gradient. For glycolysis the gradient from is defined by the module score range from -0.31 to 0.44 (smallest value being the lightest and largest the largest). For OxPhos the range is from 0.24 to 0.72. (b) Same as A for the OxPhos gene set. (c) Comparison of glycolysis and OxPhos scores in mouse single cell samples obtained using different methods. Left – module score with custom GO-based gene set used in the rest of this study; middle – score calculated using VISION based on the REACTOME database entry for 'Glycolysis' and 'Respiratory electron transport'; right – score calculated using VISION based on the custom GO-based gene set. N numbers are reported in the legend of Figure 1e. The boxplots are defined by the 25th and 75th percentiles, with the centre as the median. The minima and maxima extend to the largest value until 1.5 of the inter-quartile range (IQR) and the smallest value at most 1.5 of IQR, respectively. E – embryonic day, ZY – zygote, 4 cell – 4 cell stage, 8 cell – 8 cell stage, cMor – compacted morula, ICM – inner cell mass, Epi – epiblast, EmDisc – embryonic disc, Tb – trophoblast, Hyp – hypoblast, VE – visceral endoderm.

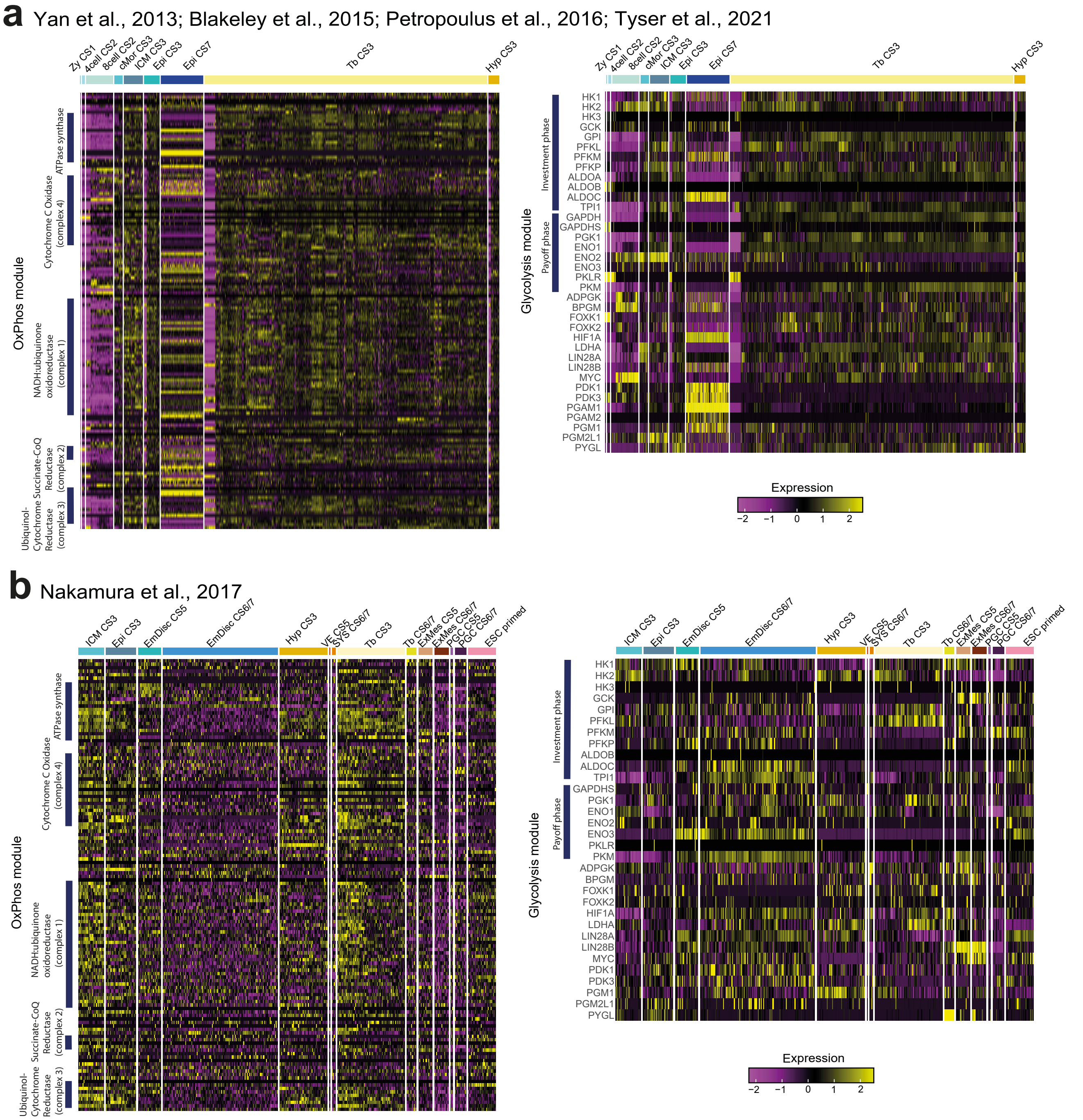

Supplementary Figure 2

(a) Heatmaps of gene expression of glycolysis and OxPhos modules of the compiled human dataset from Fig. 2b. (b) Heatmaps of gene expression of glycolysis and OxPhos modules of the compiled cynomolgus monkey dataset from Fig. 2d. CS – Carnegie stage, ZY – zygote, 4 cell – 4 cell stage, 8 cell – 8 cell stage, cMor – compacted morula, ICM – inner cell mass, Epi – epiblast, EmDisc – Embryonic disc, ESC – embryonic stem cells, Tb – trophoblast, Hyp –hypoblast, VE – visceral endoderm, SYS – secondary yolk sac, PGC – primordial germ cells, ExM – extraembryonic mesoderm.

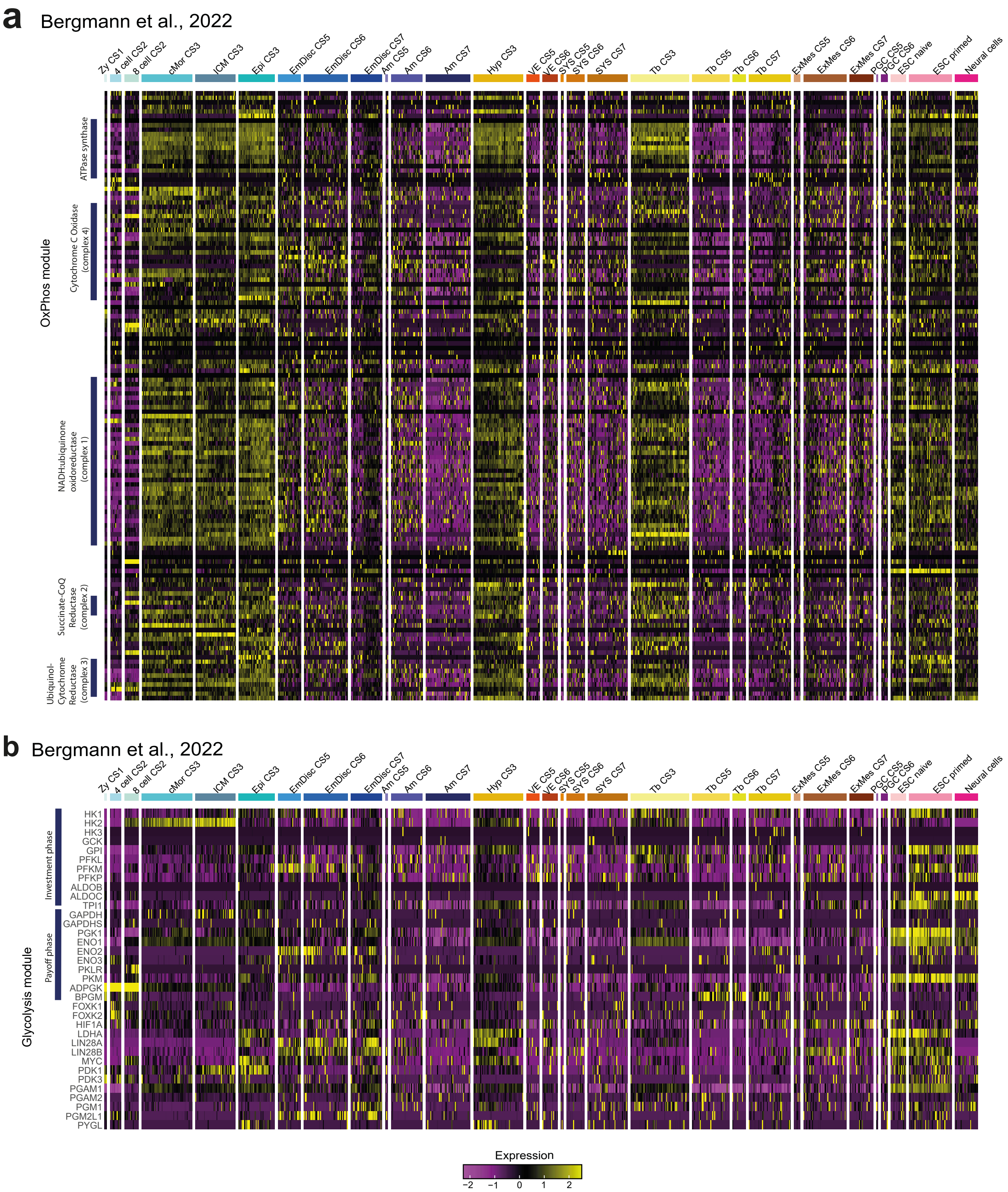

Supplementary Figure 3

Heatmaps of gene expression of the marmoset dataset from Fig. 2g for (a) the OxPhos module and (b) the glycolysis module. CS – Carnegie stage, ZY – zygote, 4 cell – 4 cell stage, 8 cell – 8 cell stage, cMor – compacted morula, ICM – inner cell mass, Epi – epiblast, EmDisc –Embryonic disc, ESCs – embryonic stem cells, Tb – trophoblast, Hyp – hypoblast, VE – visceral endoderm, AM - amnioid, SYS – secondary yolk sac, PGC – primordial germ cells, ExM – extraembryonic mesoderm.

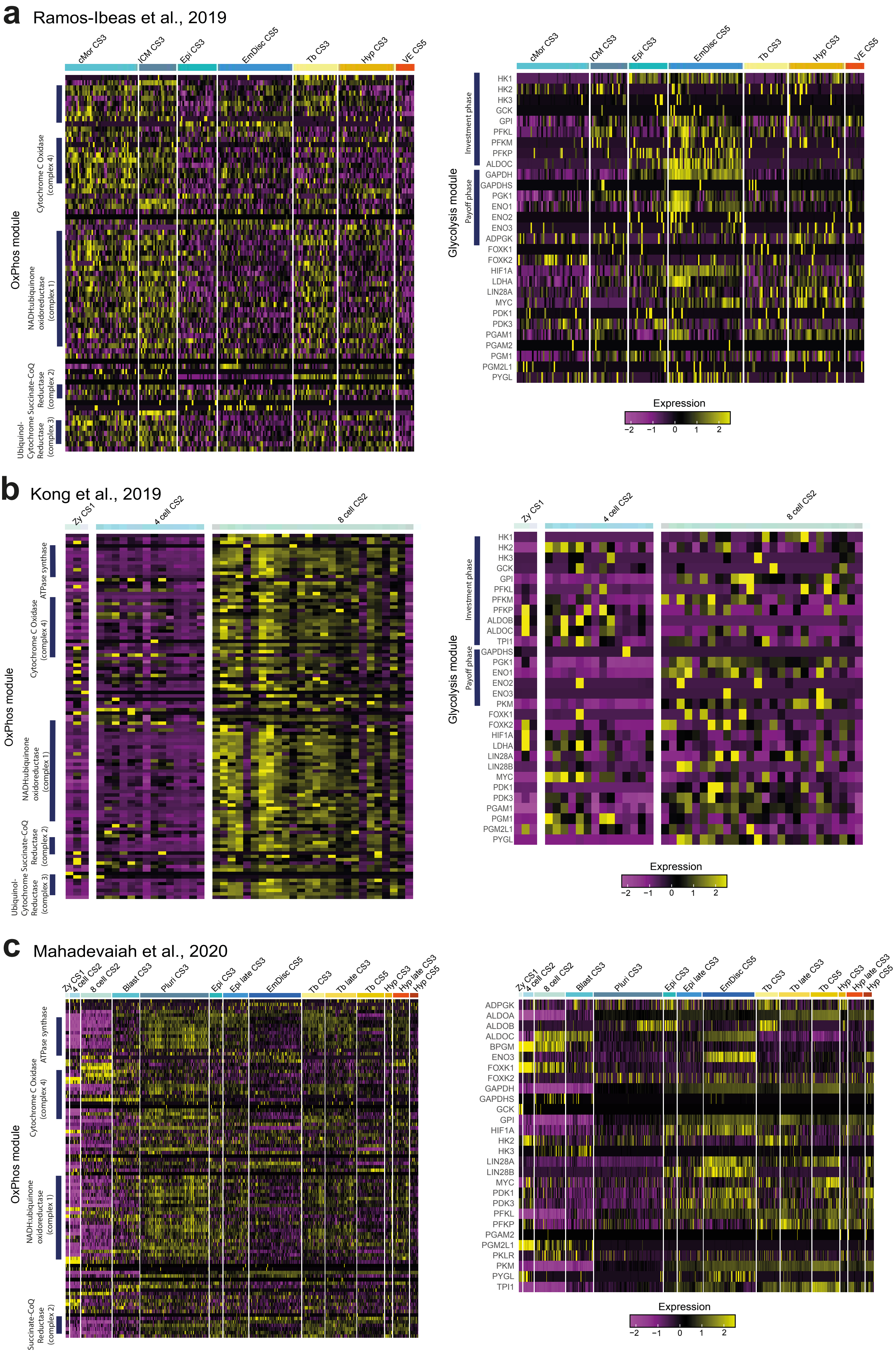
a Ramos-Ibeas et al., 2019
cMor CS3
ICM CS3
Epi CS3
EmDisc CS5
Tb CS3
Hyp CS3
VE CS5
OxPhos module
Cytochrome C Oxidase (complex 4)
NADH:ubiquinone oxidoreductase (complex 1)
Succinate-CoQ Reductase (complex 2)
Ubiquinol-Cytochrome Reductase (complex 3)
Glycolysis module
Investment phase
Payoff phase
HK1
HK2
HK3
GCK
GPI
PFKL
PFKM
PFKP
ALDOC
GAPDH
GAPDHS
PGK1
ENO1
ENO2
ENO3
ADPGK
FOXK1
FOXK2
HIF1A
LDHA
LIN28A
MYC
PDK1
PDK3
PGAM1
PGAM2
PGM1
PGM2L1
PYGL
Expression
−2 −1 0 1 2
b Kong et al., 2019
Zy CS1
4 cell CS2
8 cell CS2
OxPhos module
ATPase synthase
Cytochrome C Oxidase (complex 4)
NADH:ubiquinone oxidoreductase (complex 1)
Succinate-CoQ Reductase (complex 2)
Ubiquinol-Cytochrome Reductase (complex 3)
Glycolysis module
Investment phase
Payoff phase
HK1
HK2
HK3
GCK
GPI
PFKL
PFKM
PFKP
ALDOB
ALDOC
TPI1
GAPDHS
PGK1
ENO1
ENO2
ENO3
PKM
FOXK1
FOXK2
HIF1A
LDHA
LIN28A
LIN28B
MYC
PDK1
PDK3
PGAM1
PGM1
PGM2L1
PYGL
Expression
−2 −1 0 1 2
c Mahadevaiah et al., 2020
Zy CS1
4 cell CS2
8 cell CS2
Blast CS3
Pluri CS3
Epi CS3
Epi late CS3
EmDisc CS5
Tb CS3
Tb late CS3
Tb CS5
Hyp CS3
Hyp late CS3
Hyp CS5
OxPhos module
ATPase synthase
Cytochrome C Oxidase (complex 4)
NADH:ubiquinone oxidoreductase (complex 1)
Succinate-CoQ Reductase (complex 2)
ADPGK
ALDOA
ALDOB
ALDOC
BPGM
ENO3
FOXK1
FOXK2
GAPDH
GAPDHS
GCK
GPI
HIF1A
HK2
HK3
LIN28A
LIN28B
MYC
PDK1
PDK3
PFKL
PFKP
PGAM2
PGM2L1
PKLR
PKM
PYGL
TPI1
Expression
−2 −1 0 1 2

Supplementary Figure 4

(a, b) Heatmaps of gene expression of the pig datasets from Fig. 2i for the OxPhos module and the glycolysis module. (c) Heatmaps of gene expression of the opossum dataset from Fig. 2k for the OxPhos module and the glycolysis module. CS – Carnegie stage, ZY – zygote, 4 cell – 4 cell stage, 8 cell – 8 cell stage, cMor – compacted morula, ICM – inner cell mass, Epi – epiblast, EmDisc – Embryonic disc, Tb – trophoblast, Hyp – hypoblast, VE – visceral endoderm, Pluri – pluriblast, Blast – blastocyst.

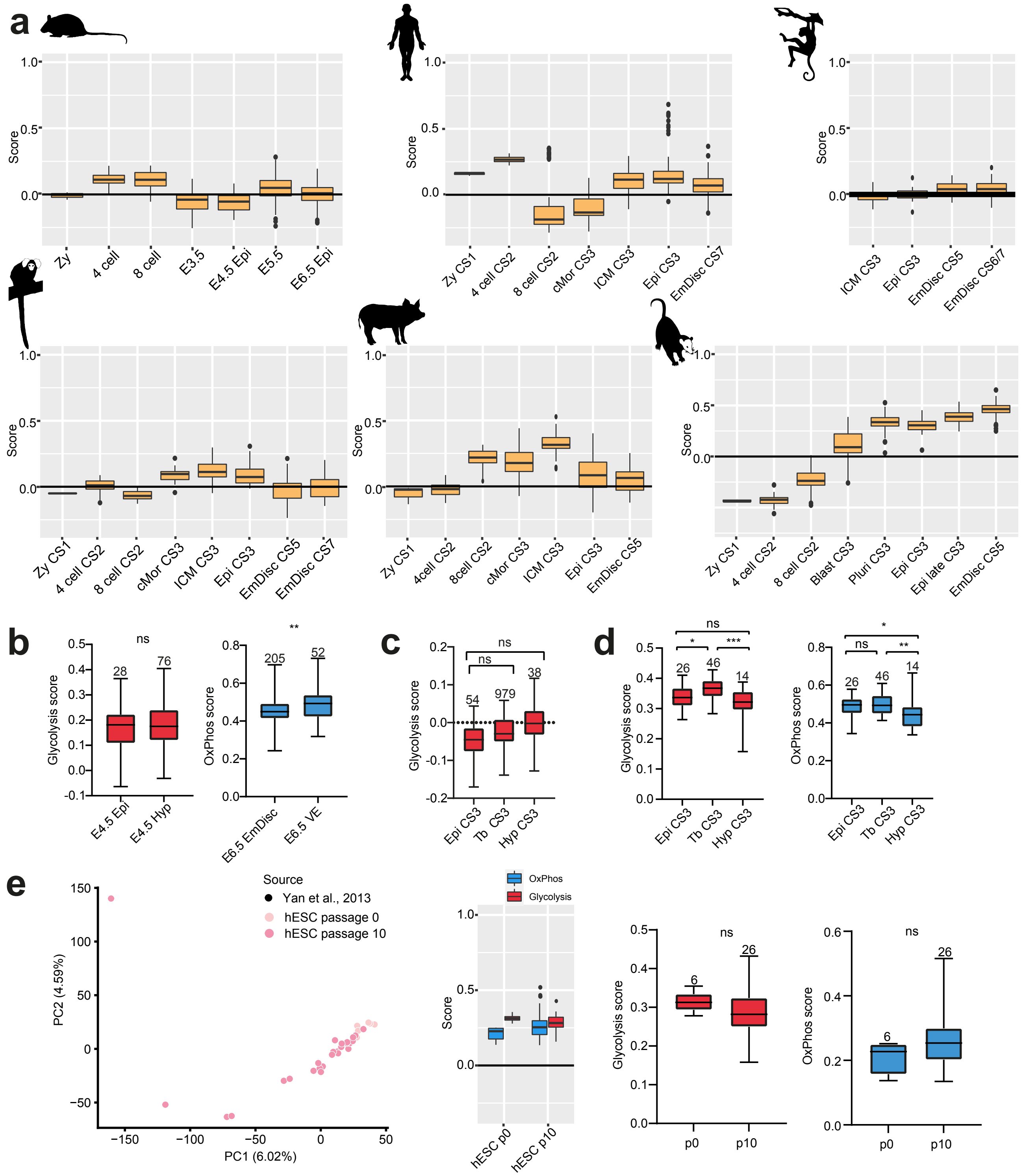
a
Score
Zy
4 cell
8 cell
E3.5
E4.5 Epi
E5.5
E6.5 Epi
Zy CS1
4 cell CS2
8 cell CS2
cMor CS3
ICM CS3
Epi CS3
EmDisc CS7
ICM CS3
Epi CS3
EmDisc CS5
EmDisc CS6/7
EmDisc CS5
4cell CS2
8cell CS2
Blast CS3
Pluri CS3
Epi late CS3
b
Glycolysis score
OxPhos score
E4.5 Epi
E4.5 Hyp
E6.5 EmDisc
E6.5 VE
c
Tb CS3
Hyp CS3
d
e
Source
Yan et al., 2013
hESC passage 0
hESC passage 10
PC1 (6.02%)
PC2 (4.59%)
OxPhos
Glycolysis
hESC p0
hESC p10
p0
p10
ns

Supplementary Figure 5

(a) Module score for pentose phosphate pathway in embryonic tissues of 6 mammalian species. The boxplots are defined by the 25th and 75th percentiles, with the centre as the median. The minima and maxima extend to the largest value until 1.5 of the inter-quartile range (IQR) and the smallest value at most 1.5 of IQR, respectively. The N numbers are presented in the legends of Figure 1e and 2c, g, e, i, k. (b) Glycolysis and OxPhos module scores for mouse samples from Figure 3A, tested using the two-sided Student's t test. The p values for glycolysis scores are as follows: E4.5 Epi vs E4.5 Hyp=0.419, for OxPhos: E6.5 EmDisc vs E6.5 VE=0.001. (c) Glycolysis module scores forhuman samples from Figure 3B, tested using Kruskall-Wallis test. P values are as follows: Epi CS3 vs Tb CS3=0.071, Epi CS3 vs Hyp CS3=0.798. (d) Glycolysis and OxPhos module score for opossum scores from Figure 3F, tested using one-way ANOVA. P values for glycolysis scores are as follows: Epi CS3 vs Tb CS3=0.027, Epi CS3 vs Hyp CS3=0.196, for OxPhos: Epi CS3 vs Tb CS3=0.834, Epi CS3 vs Hyp CS3=0.046. For (b), (c), (d) the boxplots are defined by the 25th and 75th percentiles, with the centre as the median. The minima and maxima refer to the largest and smallest values, respectively. (e) PCA and glycolysis and OxPhos scores for a hESC dataset[50]. The boxplots on the left are defined by the 25th and 75th percentiles, with the centre as the median. The minima and maxima extend to the largest value until 1.5 of the inter-quartile range (IQR) and the smallest value at most 1.5 of IQR, respectively. In the boxplots on the right the maxima and minima refer to the largest and smallest values, respectively. p0 – passage 0, p10 – passage 10. Significance tested using a two-sided unpaired Student's t-test. P values are as follows: for OxPhos=0.138, for glycolysis=0.237. All Kruskall-Wallis tests were followed by Dunn's multiple comparisons test and ANOVA's were followed by Sidak's multiple comparisons test. E – embryonic day, CS – Carnegie stage, ZY – zygote, 4 cell – 4 cell stage, 8 cell – 8 cell stage, cMor – compacted morula, ICM – inner cell mass, Epi – epiblast, EmDisc – embryonic disc, Tb – trophoblast, Hyp – hypoblast, VE – visceral endoderm.

**A** Embryo samples					
**Species**	**Reference**	**Samples**	**N**	**Conditions**	**Accession number**
Mouse *Mus musculus*	Mohammed et al., 2017	E3.5, E4.5, E.5.5, E6.5	721	*In vivo*	GSE100597
	Deng et al., 2014	Zy, 4 cell, 8 cell	51	*In vivo*	GSE45719
Opossum *Monodelphis domestica*	Mahadevaiah, et al., 2020	E1.5-E7.5: Zy-CS5	826	*In vivo*	E-MTAB-7515
Pig *Sus scrofa*	Kong et al., 2019	Zy, 4 cell, 8 cell	43	*In vivo*	GSE139512
	Ramos-Ibeas et al, 2019	E5-E10: CS3, CS4, CS5	220	*In vivo*	GSE112380
Marmoset *Callithrix jacchus*	Bergmann et al., 2022 (integrated with Boroviak et al., 2018)	CS1, CS2, CS3, CS5, CS6, CS7	824	*In vivo*	E-MTAB-9367; E-MTAB-7078
Cynomolgus monkey *Macaca fascicularis*	Nakamura et al., 2017	CS3, CS5, CS6/7	443	*In vitro* (CS3) 5% CO_2% and 5% O_2; *In vivo* (CS5-CS6/7)	GSE74767
	Ma et al., 2019	CS5, CS6, CS7	1453	Embryos recovered at E3.5 and cultured *in vitro*	GSE130114
Human *Homo sapiens*	Yan et al., 2013	Zy, 4 cell, 8 cell, CS3	57	*In vitro*, 6% CO_2, normoxia	GSE36552
	Blakeley et al., 2015	CS3	26	*In vitro*, 5% CO_2, normoxia	GSE66507
	Petropoulus et al., 2016	8 cell, CS3	1190	*In vitro*	E-MTAB-3929
	Tyser et al., 2021	CS7	147	Sample from HDBR (developed *in vivo*)	www.human-gastrula.net
	Xiang et al, 2019	CS3, CS4, CS5, CS6	547	*In vitro* from blastocysts in 10% Matrigel; 6% CO2, normoxia	GSE136447

**B** Cell culture					
**Species**	**Reference**	**Samples**	**N**	**Conditions**	**Accession number**
Mouse *Mus musculus*	Chen et al., 2016	ESCs, EpiSCs	447	5% CO_2	GSE74155
Human *Homo sapiens*	Messmer et al., 2019	naive and primed hECs	384	not reported	E-MTAB-6819
	Yan et al., 2013	hESCs	32	not reported	GSE36552

Supplementary Data 1. List of single cell RNA-seq datasets used in the study. N = number of cells.
