# Supplementary material for: A hexa-species transcriptome atlas of mammalian embryogenesis delineates metabolic regulation across three different implantation modes
Source: Nat Commun. 2022 Jun 16;13:3407. doi: 10.1038/s41467-022-30194-x (PMC9203550; doi:10.1038/s41467-022-30194-x)
Supplement: Supplementary file 4 — Reporting Summary [file 41467_2022_30194_MOESM4_ESM.pdf]

nature portfolio

Corresponding author(s): Thorsten E. Boroviak

Last updated by author(s): 2021/12/01

Reporting Summary

Nature Portfolio wishes to improve the reproducibility of the work that we publish. This form provides structure for consistency and transparency in reporting. For further information on Nature Portfolio policies, see our Editorial Policies and the Editorial Policy Checklist.

Statistics

For all statistical analyses, confirm that the following items are present in the figure legend, table legend, main text, or Methods section.

n/a	Confirmed	
☐	☒	The exact sample size (*n*) for each experimental group/condition, given as a discrete number and unit of measurement
☒	☐	A statement on whether measurements were taken from distinct samples or whether the same sample was measured repeatedly
☐	☒	The statistical test(s) used AND whether they are one- or two-sided<br>*Only common tests should be described solely by name; describe more complex techniques in the Methods section.*
☐	☒	A description of all covariates tested
☐	☒	A description of any assumptions or corrections, such as tests of normality and adjustment for multiple comparisons
☐	☒	A full description of the statistical parameters including central tendency (e.g. means) or other basic estimates (e.g. regression coefficient) AND variation (e.g. standard deviation) or associated estimates of uncertainty (e.g. confidence intervals)
☐	☒	For null hypothesis testing, the test statistic (e.g. *F*, *t*, *r*) with confidence intervals, effect sizes, degrees of freedom and *P* value noted<br>*Give P values as exact values whenever suitable.*
☒	☐	For Bayesian analysis, information on the choice of priors and Markov chain Monte Carlo settings
☒	☐	For hierarchical and complex designs, identification of the appropriate level for tests and full reporting of outcomes
☒	☐	Estimates of effect sizes (e.g. Cohen's *d*, Pearson's *r*), indicating how they were calculated

Our web collection on statistics for biologists contains articles on many of the points above.

Software and code

Policy information about availability of computer code

Data collection	No software was used for data collection as the study only utilised previously published datasets.
Data analysis	Data was analyzed using Seurat v3 and VISION in RStudio v1.3.1093. Data visualisation was performed using ggplot2 and Seurat v3. The code used to calculate and plot metabolic module score is available at a GitHub repository: https://github.com/Boroviak-Lab/HexaSpeciesMetabolomicsAnalysis.git with a DOI doi:10.5281/zenodo.5733306. Statistical analysis was performed in GraphPad Prism v8.4.2 and GraphPad Prism v9.1.0.

For manuscripts utilizing custom algorithms or software that are central to the research but not yet described in published literature, software must be made available to editors and reviewers. We strongly encourage code deposition in a community repository (e.g. GitHub). See the Nature Portfolio guidelines for submitting code & software for further information.

Data

Policy information about availability of data

All manuscripts must include a data availability statement. This statement should provide the following information, where applicable:

- Accession codes, unique identifiers, or web links for publicly available datasets
- A description of any restrictions on data availability
- For clinical datasets or third party data, please ensure that the statement adheres to our policy

The manuscript uses a collection of public datasets available under accession codes shown in Supplementary Data 1. For the purpose of generating gene lists and creating pathway diagrams, databases including Gene Ontology Knowledgebase, WikiPathways and REACTOME database were used.

Field-specific reporting

Please select the one below that is the best fit for your research. If you are not sure, read the appropriate sections before making your selection.

- [x] Life sciences
- [ ] Behavioural & social sciences
- [ ] Ecological, evolutionary & environmental sciences

For a reference copy of the document with all sections, see nature.com/documents/nr-reporting-summary-flat.pdf

Life sciences study design

All studies must disclose on these points even when the disclosure is negative.

Sample size	The sample sizes were determined based on available datasets that covered developmental stages relevant to our study.
Data exclusions	In order to generate a stage matched compendium of mammalian datasets, datapoints were excluded based on cell stages that were not part of the analysis (e.g. 2-cell stage) or lack of cell type annotation. Moreover, datapoints from human datasets were excluded based on lack of specific cell type annotation according to previous publication (Stirparo et al., 2018).
Replication	Since no data was generated during the study, replication does not apply.
Randomization	Randomization does not apply here since groups were pre-determined based on previously published metadata of given datasets.
Blinding	Blinding was not applied to this study.

Reporting for specific materials, systems and methods

We require information from authors about some types of materials, experimental systems and methods used in many studies. Here, indicate whether each material, system or method listed is relevant to your study. If you are not sure if a list item applies to your research, read the appropriate section before selecting a response.

Materials & experimental systems

n/a	Involved in the study	
x		Antibodies
x		Eukaryotic cell lines
x		Palaeontology and archaeology
x		Animals and other organisms
x		Human research participants
x		Clinical data
x		Dual use research of concern

Methods

n/a	Involved in the study	
x		ChIP-seq
x		Flow cytometry
x		MRI-based neuroimaging
